# International database of reference gamma spectra for nuclear safeguards applications

**DOI:** 10.1038/s41597-025-05455-1

**Published:** 2025-11-27

**Authors:** Dipti, L. Marian, C. Hill, A. Koning, J. J. Bland, S. R. Schwartz, J. Dreyer, D. T. Vo, J. Carbonaro, A. Berlizov

**Affiliations:** 1 Nuclear Data Section, Division of Physical and Chemical Sciences, Department of Nuclear Sciences and Applications, International Atomic Energy Agency, Vienna, A-1400 Austria; 2 Sandia National Laboratories, Albuquerque, NM 87185 US; 3 Lawrence Livermore National Laboratory, Livermore, CA 94550 US; 4 Los Alamos National Laboratory, Los Alamos, NM 87545 US; 5 Brookhaven National Laboratory, Upton, NY 11973 US; 6 Verification Technologies Section, Division of Technical and Scientific Services, Department of Safeguards, International Atomic Energy Agency, Vienna, A-1400 Austria; 7 Present Address: Department of Physics and Astronomy, Clemson University, Clemson, SC 29634 US

**Keywords:** Nuclear fuel, Experimental nuclear physics

## Abstract

Nuclear safeguards missions use gamma spectroscopy as a non-destructive measurement technique for examining nuclear materials. Despite advances in the development of detection equipment as well as software codes, one of the concerns is the lack of well-documented spectra needed to test and validate isotopic analysis codes for their applicability. To address this need, this work introduces IDB, an international database of the reference gamma spectra of uranium, plutonium and mixed oxide nuclear material samples. IDB provides access to well-characterized sets of gamma spectra described by rich metadata, including information on the sample, measurement configuration and detector specifications. These spectra are accessible in different formats, also compatible with analysis code standards, thus promoting their sustainability and maintenance.

## Background & Summary

Gamma spectroscopy is widely used in nuclear safeguards and security applications^1,2^, including nuclear proliferation and treaty verification^3,4^, as well as environmental monitoring^5^. Advances in detection technologies and computer codes have led to improvements in spectrum quality, such as better energy resolution and efficiency, hence facilitating the analysis of nuclear materials (NM) to determine their isotopic composition^6–8^. In particular, software codes for the analysis of uranium (U) and plutonium (Pu) isotopes based on gamma spectroscopy measurements have evolved significantly due to their use by safeguards agencies to monitor NM throughout the nuclear fuel cycle, from uranium conversion facilities, enrichment plants, fuel fabrication facilities to final disposal at nuclear waste sites^9–13^. In practice, these software codes and the underlying analysis algorithm are benchmarked against the spectra of well-characterized samples. Assessing the performance and applicability of these codes against certified reference materials (CRMs) and working standards in safeguard instrumentation promotes the software code development and ensures the required accuracy of the isotopic analysis results^14^.

In turn, this motivated the development of IDB, an international reference database of gamma ray spectra of U, Pu and mixed oxide (MOX) samples, an initiative led by the International Atomic Energy Agency (IAEA) in cooperation with subject matter experts from the IAEA Member States. IDB’s development was carried out under the umbrella of the IAEA Department of Safeguards Member State support task JNT A 1684: *Sustainability and maintenance of software for Pu isotopes and U enrichment*, jointly accepted by the United States (US SP), French (FRESPAS) and European Commission (EC SP) Support Programmes. The collection of reference gamma spectra in the IDB database represents contributions from US National Laboratories and IAEA safeguard’s teams. In addition to this large number of high- and low-resolution gamma spectra, a substantial part stems from the inter-comparison workshop on isotopic analysis of uranium and plutonium by non-destructive assay techniques with medium-resolution gamma spectrometers (referred to as MRGS workshop)^15,16^.

IDB is an open-source repository of reference spectral data curated by nuclear safeguards experts that was developed following the FAIR (findable, accessible, interoperable, and reusable) principles^17^ of database management. The spectra are explicitly represented by all relevant metadata attributes such as source information, measurement specifications, and detector configuration, including provenance information. The data is provided in user-friendly formats that can be accessed using standard text editors as well as specialized software tools.

## Methods

IDB contains reference spectral data of uranium, plutonium and mixed oxide samples, curated by international subject matter experts. These datasets were provided by the IAEA and the United States Support Programme while spectrum acquisitions were carried out in different laboratories across the US, the EU and in the IAEA Safeguards Analytical Laboratories in Seibersdorf. All of these spectra were validated using commercially available codes, such as FRAM (Fixed Energy Response Function Analysis with Multiple Efficiencies)^18,19^ and MGA (Multi Group Analysis)^18^ for Pu/MOX and FRAM and MGAU (Multi Group Analysis for Uranium)^18^ for U, which are widely used in the nuclear community. An overview of the reference samples used to acquire the spectra and measurement specifications is given below.

### IAEA data

The IAEA data consist of a total of 551 spectra from U, Pu and MOX materials, broadly categorized into two sets: the first set includes 161 spectra of uranium samples taken from reference standards (powders, rods, pellets) available at the IAEA’s Safeguards Analytical Laboratories in Seibersdorf; and the second set contains 390 spectra collected for the MRGS workshop, with measurements carried out in different laboratories within Europe.

The majority of the first dataset concerns four samples of powdered triuranium octoxide (U_3_O_8_) sealed in containers with an aluminium wall of 2 mm thickness. The declared uranium enrichment content in these “infinite thickness” standards used to acquire a subset of reference spectra is 0.217(5) wt%, 0.708(1) wt%, 3.105(3) wt%, and 19.820(10) wt%, whereby, the numbers in the parentheses represent 2*σ* uncertainties in the last significant digits of ^235^U isotope abundance. These samples were measured unshielded and shielded by 7.85 gm/cm^3^ steel with thicknesses of 4 mm, 8 mm, 12 mm and 16 mm.

**Fig. 1:**
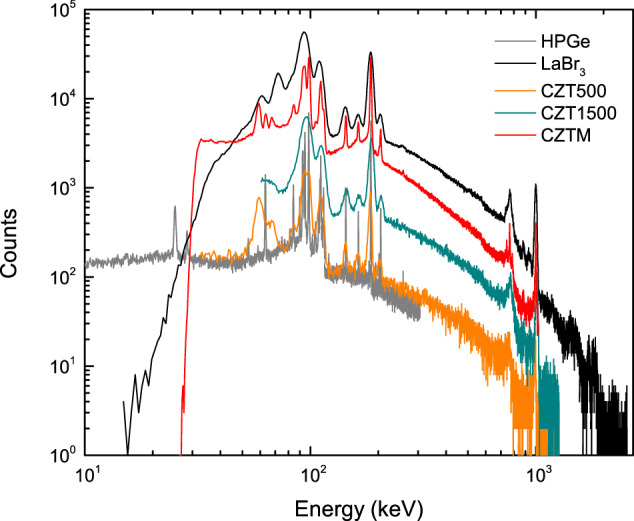
Gamma spectra of unshielded sample with ^235^U isotope abundance of 3.105(3) wt%, measured with five detectors for a live time of 500 s.

The detectors used for these measurements were three models of cadmium zinc telluride (CZT) with different geometries and sizes: two quasi-hemispherical crystals 500 mm^3^ (referred as CZT500) and 1500 mm^3^ (CZT1500) in size, four rectangular pixelated crystals of 4000 mm^3^ each (CZTM), and energy resolutions: with full-width at half maximum (FWHM) at the 186 keV peak of 5.52 keV (CZT500), 9.1 keV (CZT1500), and 2.4 keV (CZTM). Spectrum measurements were also taken using a lanthanum bromide (LaBr_3_ (Ce)) detector with cylindrical geometry ($\varnothing$2′′  × 0.5′′), which has broad gamma-ray energy coverage and lower resolution of 10.13 keV FWHM compared to CZT models. All detectors were housed inside tungsten shielding. The CZT500, CZT1500 and LaBr_3_ detectors were coupled to an MCA-527 multi-channel analyzer (GBS Elektronik). The CZTM is operated by an application specific integrated circuit (ASIC) built into the detector module, with spectral information transmitted via USB to the operating PC. High-resolution spectra measurements also were carried out using a lead-shielded high-purity germanium (HPGe) detector with planar geometry, dimensions of 1000 mm^2^ × 15 mm and FWHM of 0.66 keV. The distance between source and detector was adjusted to 0.5 cm for the CZT500 and CZT1500, 2 cm for the LaBr_3_ and CZTM, and 3.5 cm for the HPGe detectors. Spectra were collected for live time (LT) covering the range from 300 s to 4 000 s. Figure 1 shows the spectra recorded for the sample with ^235^U isotope abundance of 3.105(3) wt%, over a LT of 500 s with all detectors described above, and demonstrates the differences in energy resolution and coverage range of the detectors.

Further acquisition of the reference spectra was carried out using the uranium dioxide (UO_2_) samples, including PWR and BWR fuel rods^20^ with 0.83 mm Zircaloy cladding, with a low enrichment level between 0.716 wt% and 3.795 wt%. The fuel rods,  ~9-10 mm in diameter, were observed by the detector from the side, whilst the source to detector distance was set at about 3 cm. Additionally, four samples consisting of 5 pellets ($\varnothing$10.612 mm  × 12.204 mm) were measured inside a 3 mm thick high-density polyethylene (HDPE) holder. Spectrum acquisitions were carried out using the LaBr_3_ detector (with associated electronics as described above) for LTs ranging from 150 s – 3 600 s.

As for the MRGS data, 210 reference spectra were collected on the powdered U_3_O_8_ samples, with spectrum acquisitions carried out at the Karlsruhe site of the European Commission’s Joint Research Centre (EC-JRC Karlsruhe) and at the Institute for Radiation Protection and Nuclear Safety (IRSN) (now known as ASRN, The Nuclear Safety and Radiation Protection Authority), France. The EC-JRC Karlsruhe measurements belong to a set of five certified low-enriched uranium samples^21^, for which spectra were acquired using the CZT500, CZT4000 and LaBr_3_ detectors with a LT of 100 000 s without any shielding. Five additional spectra from these samples were measured using a coaxial HPGe detector with a 1 mm poly (methyl methacrylate) (PMMA) filter. The IRSN data consist of 155 uranium spectra from its ten standard samples^22–31^ for low to highly enriched ^235^U (0.71 wt% – 89.3 wt%). Spectra were collected unshielded and shielded over LTs of 1 000 s and 86 400 s.

In the case of Pu, 92 spectra from well-characterized pellets of plutonium oxide (PuO_2_) samples were collected at EC-JRC Karlsruhe. The source materials for these measurements consisted of four samples of the certified nuclear reference material CBNM NRM 271^32^ and four of the PIDIE^33^ series. The measurements were taken using the same detectors as for the U samples with cadmium (Cd) and steel absorbers. Another 32 spectrum acquisitions were performed for powdered PuO_2_ samples^34^ at the Safeguards Analytical Laboratories using the CZT500, CZT4000 and LaBr_3_ detectors with Cd/steel absorbers. Additionally, the same detectors were used to acquire 56 spectra for four MOX samples^35–38^ with a U/Pu ratio between 1.08% and 14.48 % using the same detectors. The spectra were recorded with Cd or steel or both absorbers between the sample and the detector. More details on the MRGS measurement configuration and data analyses can be found in References^15,39^.

### US SP data

The US Support Program provided 1 040 reference gamma spectra from U, Pu, and MOX samples. Spectrum acquisitions were performed at Los Alamos National Laboratory (LANL) and Lawrence Livermore National Laboratory (LLNL), included the Pu and MOX spectra measured at LANL for the MRGS exercise.

Nearly all uranium spectra, 598 out of 601, are associated with powder U_3_O_8_ samples with ^235^U isotope abundance ranging from 0.3 wt% to 93.3 wt%. Five types of spectrometers, among them CdTe, CZT, LaBr_3_, including those used for both low-resolution (NaI) and high-resolution (HPGe) gamma spectroscopy, were employed for spectrum acquisitions. The CZT detector used in these measurements features a co-planar geometry (CPG), whose energy resolution was significantly better compared to the standard hemispherical type. Detector specifications and operating parameters used for these measurements are listed in Table 1. The plutonium set includes 415 spectra collected from several samples measured at LANL and LLNL with the HPGe, LaBr_3_ and CdTe detectors. Additionally, 24 high-resolution spectra for MOX samples with a U/Pu ratio between 0.32% and 37.82% were acquired at LANL with HPGe detectors.

**Table 1:** Detector specifications and parameters used for uranium spectrum measurements performed at the US national laboratories LANL and LLNL.

Detector type	Size/geometry	Energy range (keV)	FWHM (keV)	LT (s)	No. of spectra
CdTe	10 mm × 10 mm × 1 mm Peltier cooled	0–250	8.37	678–881	8
CZT	15 mm × 15 mm × 15 mm CPG	0–250	1.56	300	8
HPGe	Planar	0–310/0–1200	0.65/0.73	314–3600	541^*a*^
	Coaxial	0–1024^*b*^/0–1024^*c*^/0–3000^*d*^	0.99/1.3/1.34		
	BEGe/Falcon5000	0–600	0.93		
LaBr_3_	$\varnothing$25 mm × 38 mm/Unknown	0–250/0–1600	10.67/11.59	300, 600	36
NaI	$\varnothing$25 mm × 51 mm	0–250	19.52	300	8

The FWHM (keV) represent energy resolution at 186 keV peak.

^*a*^Among the 541 spectra, three spectra belong to samples with unknown isotopic abundance and were collected for longer acquisition live times of 68 396 s, 78 294 s, and 81 758 s;

^*b*^about 25% relative efficiency closed end coaxial detector;

^*c*^120% relative efficiency coaxial detector ORTEC GEM;

^*d*^ORTEC Detective.

## Data Records

The reference gamma spectral datasets are available in the NDS repository^40^, an IAEA institutional repository for nuclear data. All datasets are accessible as a downloadable archive, IDB-v2024-01, and are released under the CC-BY-4.0 license. Each spectrum in the repository^40^ is described by rich metadata providing details about the source material and measurement setup, along with detector specifications and shielding configuration, associated spectrum metadata and source information, which in turn includes certified data on U and Pu isotopic compositions, such as uncertainties, production and material characterization information, sample design and physical dimensions. The material metadata contains information on the isotopic composition at the time of spectrum acquisition, which is corrected for decay based on the certified data, including source information. The detector metadata includes details of the detector type and geometry, as well as its size, analyzer name and gain, number of channels; FWHM of the 186 keV or 208 keV peaks for U and Pu measurements, respectively; detector energy range, and source-to-detector distance. Further measurement specifications, such as attenuating materials and associated electronics, are provided in measurement metadata. Spectrum metadata includes date of acquisition, real time, live time, dead time, input count rate, total number of counts, along with the information about the data provider.

**Fig. 2:**
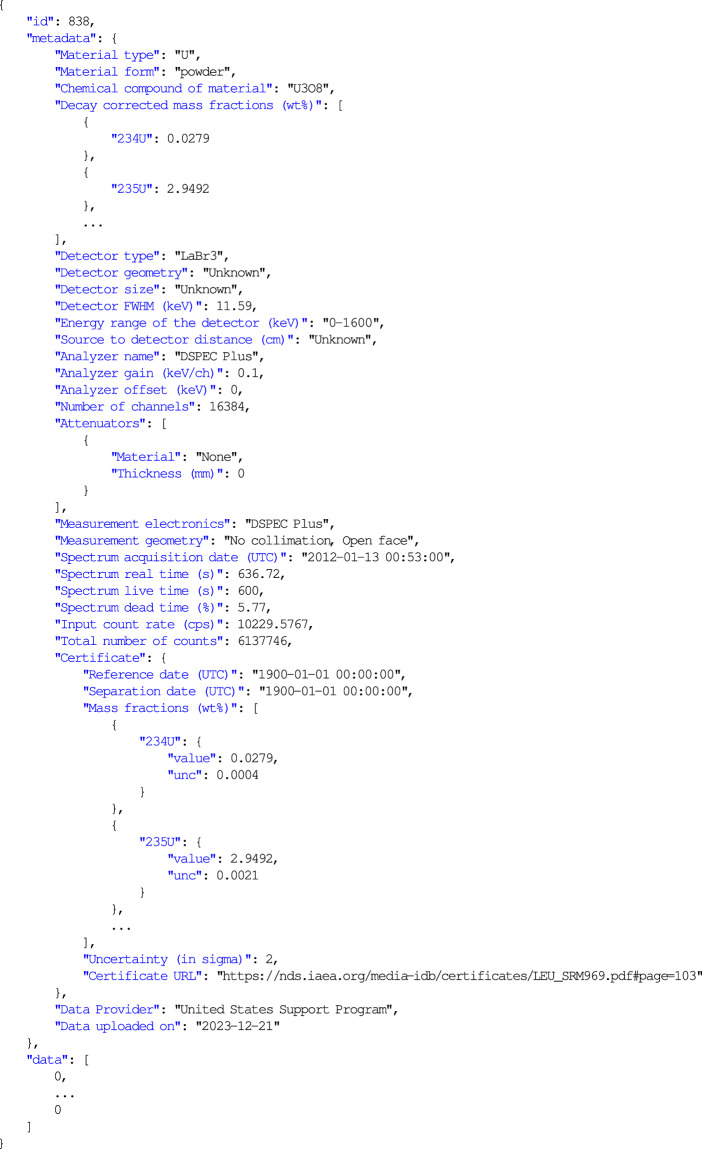
An example JSON dataset file for download.

The IDB spectra are stored using the IAEA SPE file format, which is one of the standard data exchange format^41,42^ for storing and managing spectral data, particularly in the context of nuclear spectroscopy. It’s a block-structured, ASCII text file, meaning it’s human-readable and can be opened and analyzed using standard text editors as well as compatible with various isotopic analysis codes. The SPE format is open and non-proprietary. An example spectrum in the SPE file format is given in Table 2, along with the explanation of the metadata and spectrum data as counts per channel in the $DATA block. Additionally, a JSON file (JavaScript Object Notation, a type of file format) for an example spectrum is presented in Fig. 2, which provides spectrum data and associated metadata in key-value pairs. Isotope abundance uncertainties of the reference data are given in relative units in the SPE file, while in the JSON format they correspond to absolute units. This also applies to the effective abundance of plutonium (^240^Pu_eff_), calculated as:

$${{}^{240}{\rm{Pu}}}_{{\rm{e}}{\rm{f}}{\rm{f}}}({\rm{w}}{\rm{t}}{\rm{ \% }})=2.5{2}^{238}{\rm{P}}{\rm{u}}{+}^{240}\,{\rm{P}}{\rm{u}}+1.6{8}^{242}{\rm{P}}{\rm{u}}$$

**Table 2:** An example spectrum in SPE file format with explanation of the metadata.

Block	Example	Description
$APPLICATION_ID	IDB v.1.0	Application name and version, separated by space
$SPEC_REM	Material type: U	This block contains information on the type of nuclear material (U/Pu/MOX), including its physical form and chemical composition, as well as its decay-corrected U and Pu isotopic composition. It also provides measurement setup information, such as detector specifications (including geometry and size), and the multichannel analyzer type.
	Material form: powder	
	Material compound: U3O8	
	U isotopics:	
	234U 1.0178	
	235U 93.1109	
	236U 0.4362	
	238U 5.4347	
	Detector: HPGe (Geometry: Planar, Size: Unknown)	
	MCA: DigiDart	
$DATE_MEA	06/14/2004 18:22:00	Date and time of the measurement
$MEAS_TIM	68396 76521	Live time (s) and real time (s) of the measurement as integers
$DATA	0 8191	First channel (0) Last channel (Number of channels minus 1)
	0	Counts in channel 0
	0	Counts in channel 1
	…	…
$ENER_FIT	0.00000 0.12500	Offset (keV) and amplification gain (keV/ch)
$PRESETS	Live Time (sec)	Start and end of spectrum acquisition live time (s)
	68396	End time as an integer
	0	Start time (0)
$RT	76521.234	Real time (s) as a real number
$DT	8125234	Dead time (ms) as an integer
$ISO_DECL_U	SMPLNAM: IDB-1	This block is included to provide information on the reference data for U or MOX samples, when available. • SMPLNAM: Sample name, IDB- prefix followed by an integer representing the spectrum ID • SEPDATE: Separation date of the nuclear material, if unknown, indicated by 01/01/1900 00:00:00 • UXXX and DUXX: Isotopic abundance (in wt%) and relative uncertainties for U isotopes
	SEPDATE: 01/01/1900 00:00:00	
	U234: 1.0178	
	DU234: 0.0983	
	U235: 93.1109	
	DU235: 0.0055	
	U236: 0.4362	
	DU236: 0.0688	
	U238: 5.4347	
	DU238: 0.0478	
$ISO_DECL_PU	SMPLNAM: IDB-145	This block contains information for reference data on Pu or MOX samples, when available. SMPLNAM and SEPDATE are described in $ISO_DECL_U, while new entries are explained below: • PUXXX and DPUXX: Isotope abundances (in wt%) and relative uncertainties for Pu isotopes • AM241 and DAM241: ^241^Am abundance (in wt%) and its relative uncertainty • DATE: Reference date for Pu isotopic abundances as given in the certificate • PU240EFF and DPU240EFF: ^240^Pu effective abundance and its relative uncertainty
	SEPDATE: 01/01/1900 00:00:00	
	PU238: 0.01448	
	DPU238: 3.4722	
	PU239: 93.778	
	DPU239: 0.0055	
	PU240: 5.8618	
	DPU240: 0.0887	
	PU241: 0.2798	
	DPU241: 0.1787	
	PU242: 0.0658	
	DPU242: 2.4316	
	AM241: 0.0198	
	DAM241: 1.0101	
	DATE: 10/28/1988 00:00:00	
	PU240EFF: 6.0086	
	DPU240EFF: 0.2130	
$ADC:	8192	Total number of channels
	0	First channel (0)
	8191	Total number of channels minus 1

## Technical Validation

All data acquisitions were performed using measurement systems (detectors, electronics, software) that went through a formal testing and validation processes to ensure the correctness and completeness of the measurement data. All instruments employed for acquiring the IAEA dataset belonged to the IAEA pool of equipment officially authorized for use in international safeguards verifications. Spectrum acquisitions were conducted in laboratory environments, with well-controlled radiation background and ambient conditions.

The validity of the IDB data is further vouchsafed by the quality of samples utilized for collecting the spectra; these represented either certified reference materials or secondary (working) standards, whose characterization had been performed by ISO-accredited laboratories using best analytical practices and metrologically certified methods, techniques and equipment. The relevant sample information (such as isotopic abundances, elemental composition, impurity contents, production/separation/characterization dates, design drawings etc.) is part of the metadata associated with each IDB spectrum.

In the course of data preparation, the integrity and traceability of the data were assured through the use of checksums and by documenting all steps undertaken towards production of the final dataset. The preparation process included data review and validation performed by subject matter experts. An important element in the process was the use of well-established software codes, such as FRAM^43^, MGA^44^ and MGAU^45^, for processing the spectra and comparing the derived isotopic abundances against certified values. The codes’ performance was demonstrated and their applicability supported by the results of the inter-comparison exercise and numerous studies published (see^15^ and references therein).

## Usage Notes

In addition to the repository data, our data are also available at https://nds.iaea.org/idb/. The database is built on a relational database management system backend and searchable via an online user interface (UI) and API that allows data to be retrieved by querying over a set of metadata attributes, accompanied by tools for data visualization. The UI design, data representation, search capabilities and other functionalities provided by the IDB web-page were extensively tested by a worldwide group of subject matter experts prior to public release of the data. All versions of the database can be downloaded from the versions page at https://nds.iaea.org/idb/versions.

**Fig. 3:**
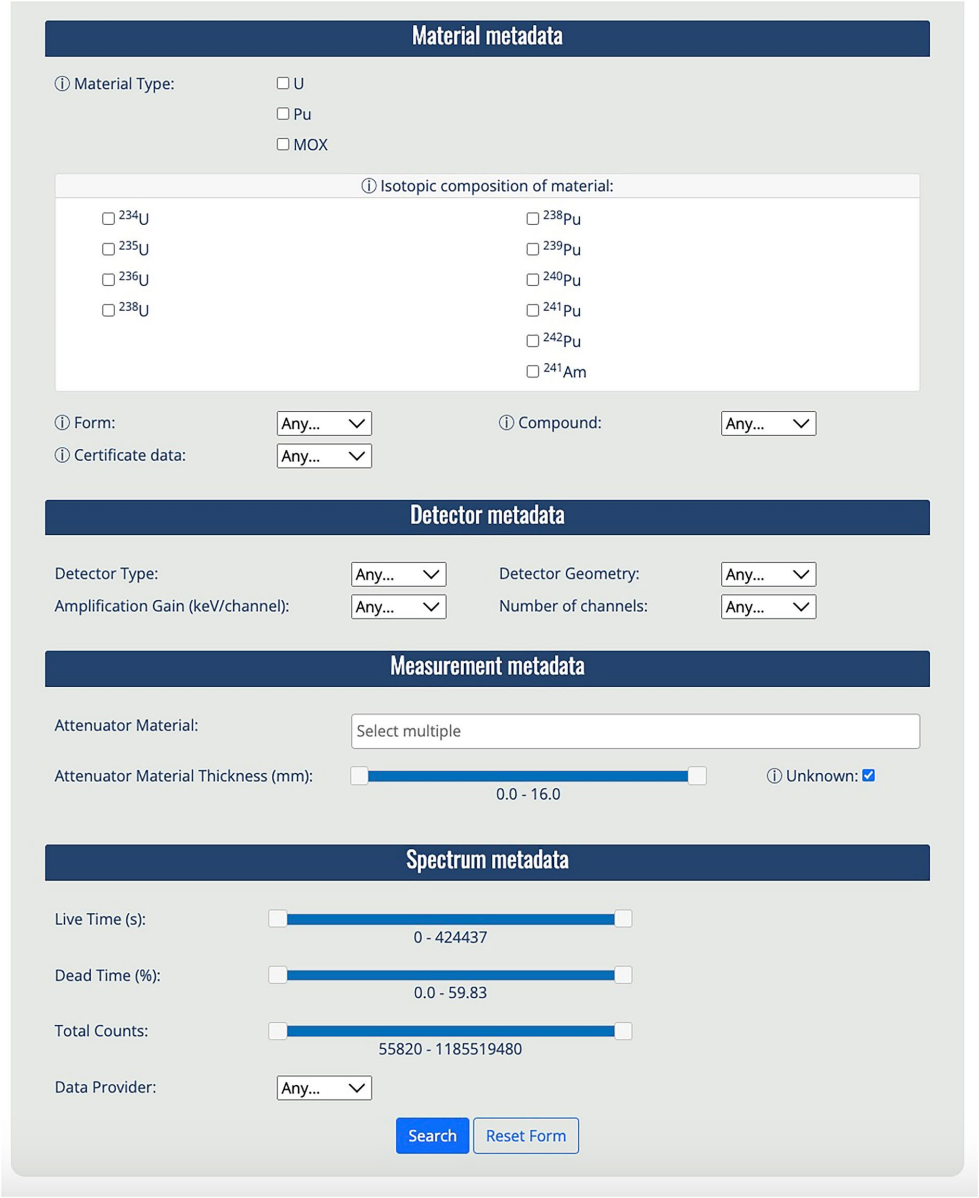
The IDB browser-based search interface available online at https://nds.iaea.org/idb/search/.

At the time of this writing, it contains 1 591 datasets, described with rich metadata and searchable via a browser-based interface. The online search form for interacting with the database is shown in Fig. 3. The default search returns all spectra in the database, but results can be refined by filtering on a set of associated metadata attributes. As shown in Fig. 3, users can select their preferences from the options available through the UI, thereby simplifying their interactions with database (SQL generated behind the scenes). Examples include the retrieval of gamma spectra associated with U, Pu or MOX materials or based on the isotopic abundance of U or Pu isotopes or the U/Pu ratio in the case of MOX samples. Other search options include the selection from the drop-down menu of physical and chemical composition of the material, attenuator material and detector metadata, including type, geometry, amplification gain and number of channels. The search can be further refined to filter on the spectrum metadata, e.g., live time, dead time and input count rate. Additionally, the database can also be queried based on data provenance, allowing users to retrieve spectra from a specific data provider.

**Fig. 4:**
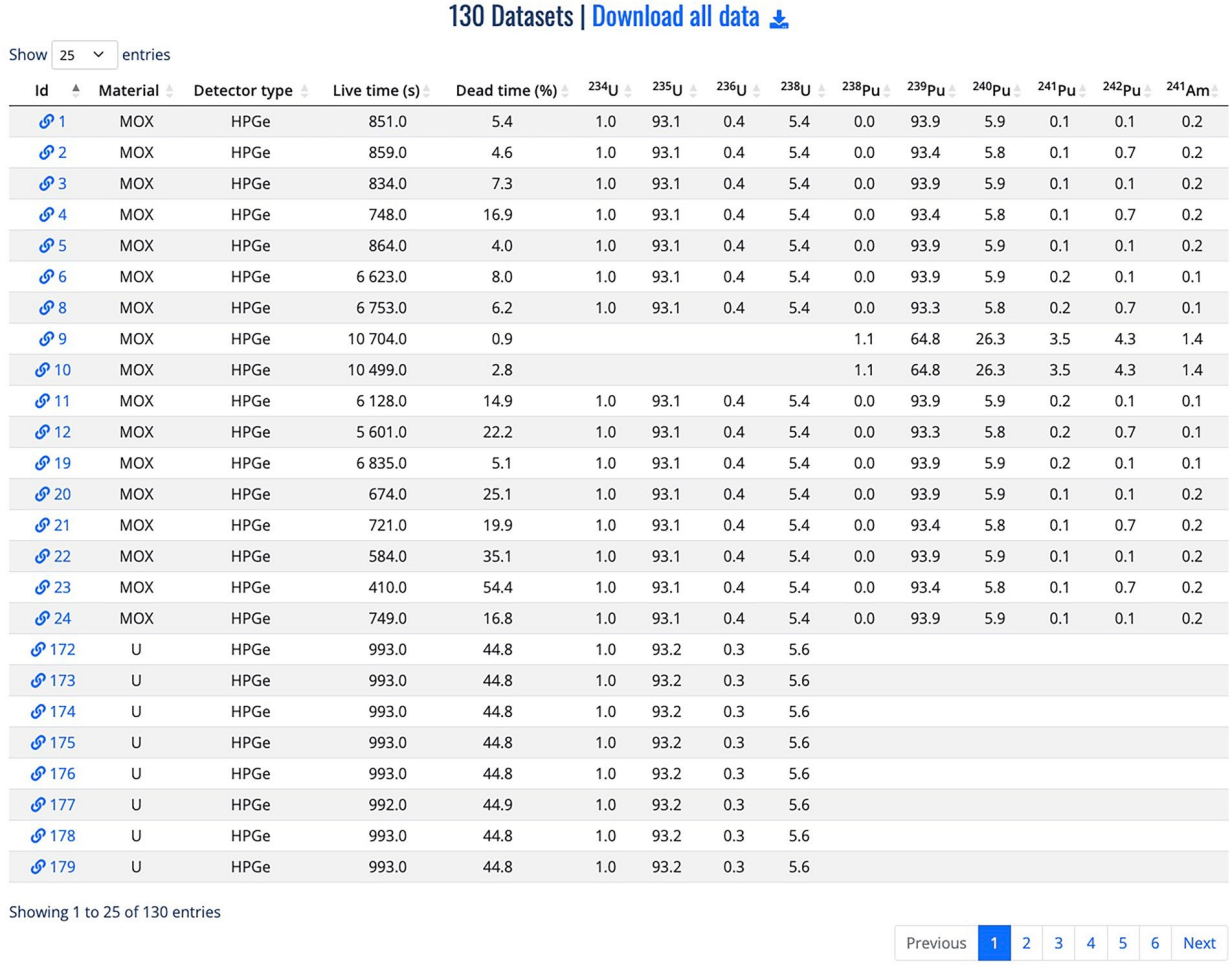
An example of database query results, showing the list of spectra identified by their ID, along with some relevant metadata and sorting functionality.

**Fig. 5:**
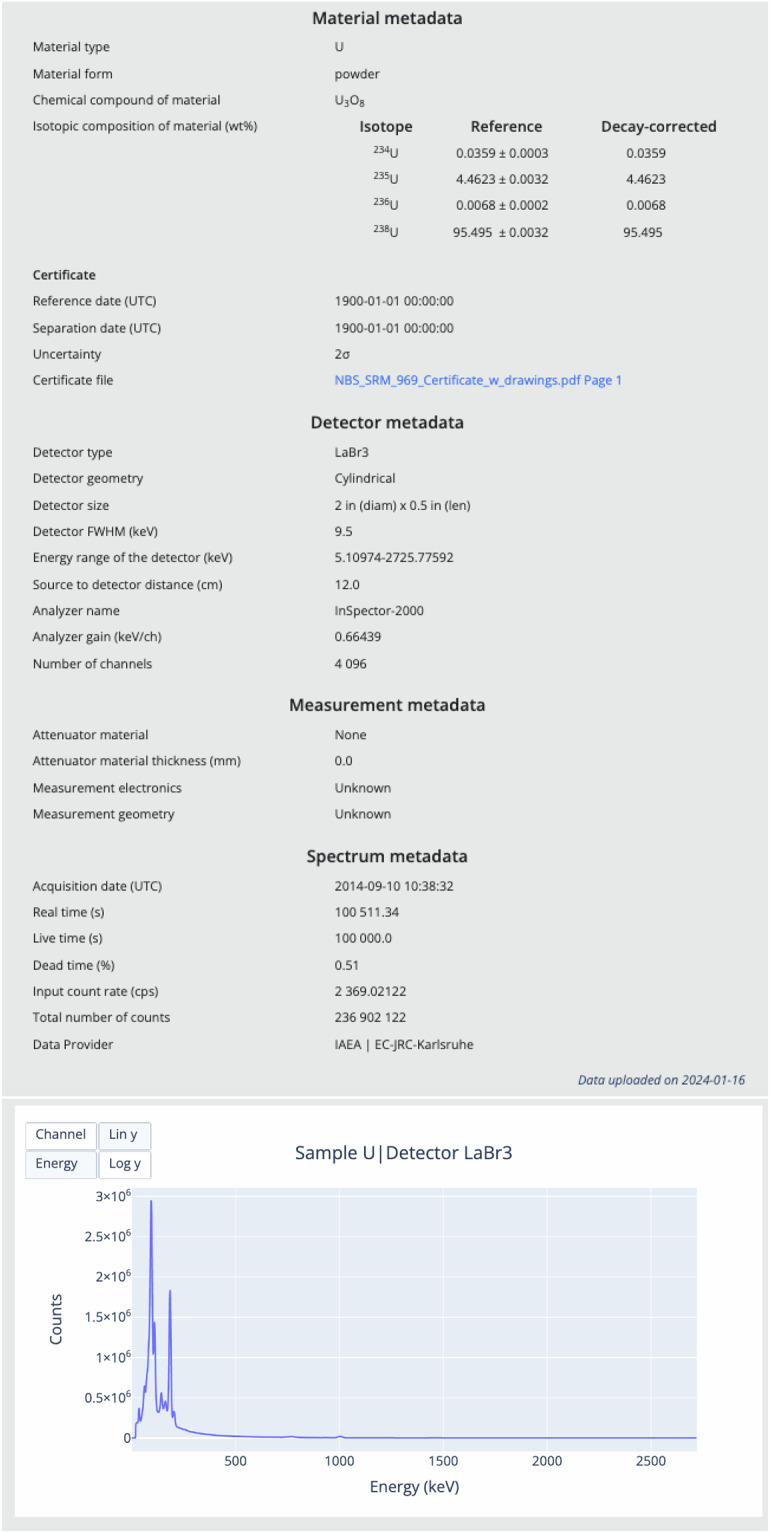
An example showing detailed information along with a graphical representation for a spectrum with primary key ID 1256. Available online at https://nds.iaea.org/idb/spectra/1256.

Spectra corresponding to the search query are displayed in a paginated list on the search results page and can be downloaded as a compressed archive, as shown in Fig. 4 for spectra acquired with the HPGe detector for a specified uranium enrichment range (63.50 ≤^235^U ≤ 93.28). Users can visualize all the details of a given spectrum, as well as an interactive graphical rendering of the spectrum data, as illustrated in Fig. 5 for a dataset with primary key ID 1256. To enable further or local processing, users can download individual spectra into text files in different formats (SPE, JSON and CSV) via the web interface.

**Fig. 6:**
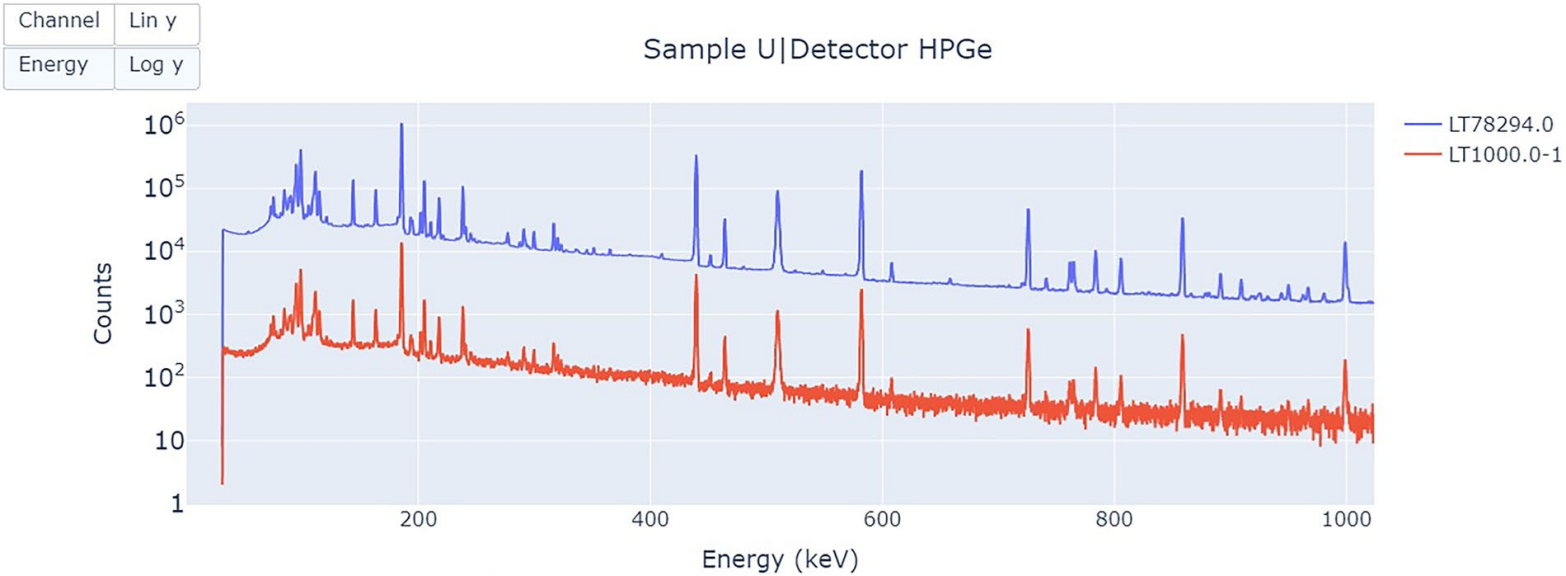
Gamma spectra of a uranium sample measured with the HPGe detector for a live time of 78294 s (blue), along with downgraded spectra generated for shorter live time of 1000 s (red). Resource available online at https://nds.iaea.org/idb/spectra-generator.

In practice, in the field, there may be time constraints on data collection. To facilitate spectral analysis for determining the abundance of U and Pu isotopes, an additional online tool generates downgraded spectra from a reference spectrum and anticipates on-site conditions. An example of downgraded gamma spectra of a uranium sample for a shorter livetime of 1 000 s, corresponding to a typical field time, is shown in Fig. 6. It is generated from a reference spectrum measured with the HPGe detector for a live time of 78 294 s.

To further assist with the automatic data processing used in isotopic abundance software codes, an API extends the capabilities of the IDB user interface to allow data to be retrieved in a standardized programmatic way. The database can be queried and accessed using an HTTP GET request: a list of parameters provided as key=value pairs, each separated by &, via https://nds.iaea.org/idb/api/search?parameter1=value1&parameter2=value2&…. For instance, to search for a specific material composition, the parameter key isotope can be be used with a unique identifier. Table 3 lists all isotopes in IDB along with their parameter keys. Thus, for example, all spectra constituting the ^238^Pu isotope (parameter key : isotope1) can be retrieved in a standard output format via https://nds.iaea.org/idb/api/search?isotope1=on. More information about the API is available online at https://nds.iaea.org/idb/api-documentation. The default API response is in JSON format while the compressed archive of text files in the SPE format can be accessed by adding the argument format=SPE in the above URL.

**Table 3:** Isotopes in IDB.

Isotope ID	Isotope
isotope1	^238^Pu
isotope2	^239^Pu
isotope3	^240^Pu
isotope4	^241^Pu
isotope5	^242^Pu
isotope6	^234^U
isotope7	^235^U
isotope8	^236^U
isotope9	^238^U
isotope10	^241^Am

## Data Availability

The data were acquired using commercially available codes, and the spectra were validated by performing their analysis with codes well established and widely used in the international community. All of these codes are commercially available and relevant URLs are provided in the Methods section.
